# Magnetic resonance imaging of pure ovarian dysgerminoma: a series of eight cases

**DOI:** 10.1186/s40644-021-00427-1

**Published:** 2021-10-28

**Authors:** Laura Maria Cacioppa, Federico Crusco, Francesco Marchetti, Michele Duranti, Matteo Renzulli, Rita Golfieri

**Affiliations:** 1 Department of Radiology, IRCCS Azienda Ospedaliero-Universitaria di Bologna, Via Albertoni 15, Bologna, Italy; 2 Division of Radiology 1, S. Maria della Misericordia Hospital, Perugia, Italy

**Keywords:** Ovarian neoplasms, Dysgerminomas, Magnetic resonance imaging, Diffusion MRI, Computed tomography

## Abstract

**Background:**

Imaging findings have a prominent role in early and correct identification of ovarian dysgerminoma, the most common ovarian malignant germ cell tumor (OMGCT). Despite Computed Tomography (CT) is widely used, Magnetic Resonance Imaging (MRI) has proved to be superior in adnexal masses characterization. Limited data and small series are available concerning MRI aspects of dysgerminoma.

**Case presentation:**

From January 2012 to December 2018, a database of solid ovarian masses was retrospectively reviewed. Eight patients with histologically proven pure ovarian dysgerminoma and complete imaging available were identified and analyzed. Imaging findings were evaluated separately by two radiologists expert in female genito-urinary MRI.

**Conclusions:**

MRI findings of a lobulated, purely solid, encapsulated mass with hyper-intensity of lobules and hypo-intensity of septa on T2w images contribute to differentiate dysgerminomas from other ovarian neoplasms.

## Background

Dysgerminoma, the ovarian counterpart of testicular seminoma, is the most common OMGCT, accounting for 1-2% of primary ovarian neoplasms and 32.8-37.5% of all OMGCTs [1]. Compared to epithelial cancers, OMGCTs are less invasive and have a favorable prognosis following surgery, even in the most advanced cases [1–3]. In particular, the prognosis of ovarian dysgerminomas is excellent, with a 5-year survival rate of almost 100% [4]. Therefore, an early and correct diagnosis of these neoplasms is of great importance.

The onset symptoms include acute or chronic pain, abdominal distension, menstrual irregularities and infertility. This tumor is commonly associated with elevated levels of lactate dehydrogenase (LDH) and occasionally with elevated beta human chorionic gonadotrophin (beta-HCG). Elevations in alpha-fetoprotein (AFP) and Cancer Antigen 125(CA-125) are less common [5]. Differently from several other ovarian neoplasms, no specific laboratory tumor markers are correlated with this histotype. In vast majority of cases, dysgerminomas are incidentally diagnosed in asymptomatic womens [1–3].

The main imaging findings of dysgerminoma include a well-marginated solid lobulated mass with fibrovascular septa. However, this feature is unspecific for dysgerminoma [6]. Despite CT is widely used in detecting female pelvic masses, MRI has prove to be superior in their characterization [7]. However, at the state of art, limited data are available concerning MRI characteristics of dysgerminoma, furthermore described in small sample series [8–10].

The aim of the present study is to describe MRI characteristics of dysgerminoma in a consecutive series of patients, with proven histological diagnosis of this rare tumor.

## Case presentation

### Materials and methods

From January 2012 to December 2018, a database of solid ovarian masses histologically proven was retrospectively reviewed and imaging characteristics of ovarian dysgerminomas confirmed at histology were collected. Eight patients with ovarian dysgerminoma and complete imaging were identified and analyzed.

Demographic, clinical and laboratory data of these patients are reported in Table 1.

**Table 1:** Demographic,clinical and laboratory data of study population

Patients(n)	1	2	3	4	5	6	7	8
**Age (yrs)**	18	15	23	34	19	20	39	13
**Symptoms/ signs**	Chronic pelvic pain Infertility	Acute pelvic pain Menstrual irregularities	No symptoms	Infertility Acute pelvic pain Palpable mass	Menstrual irregularities	Chronic pelvic pain Abdominal distention	Abdominal distention Palpable mass	Chronic pelvic pain Palpable mass
**Markers values**^**a**^	LDH: 3160 AFP: 0 B-HCG: 12 CA125: 30	LDH: 2360 AFP: 0 B-HCG: 0 CA125: 5	LDH: 318 AFP: 0 B-HCG: 0 CA125: 187	LDH: 7000 AFP: 0 B-HCG: 0 CA 125: 10	LDH: 3160 AFP: 0 B-HCG: 12.4 CA 125: 0	LDH: 130 AFP: 0 B-HCG: 0 CA125: 32	LDH: 118 AFP: 18 B-HCG: 0 CA125: 18	LDH: 360 CA125: 12 B-HCG: 0 CA125: 10

^a^*Normal values: LDH: 120-150 UI/ml, AFP: 0-20 ng/ml, beta-HCG < 0.1 ng/ml, CA-125: 0-35 UI/ml*

The median age was 22.6(13-39) years. One patient was asymptomatic; the remaining patients demonstrated the following symptoms: chronic pelvic pain (*n* = 3; 38%), infertility (*n* = 2; 25%), irregular menstruation (*n =* 2; 25%), abdominal distension (*n =* 2; 25%), acute pelvic pain (*n* = 3; 37.5%). Four patients (50%) complained of two of those symptoms. In 3 patients (37.5%), a palpable mass was detectable at physical examination.

One patient was pregnant at the time of diagnosis. Seven patients were nulliparous (87.5%), one was multiparous (12.5%). None was previously submitted to abdominal-pelvic surgery. One patient had uretero-pelvic junction syndrome with right hydronephrosis.

Serum tumor markers showed an elevated LDH value in 6 patients (75%), elevated CA-125 in one patient (12.5%), elevated beta-HCG in one case (12.5%), normal values of AFP in all cases.

After the admission, all the patients were submitted to trans-abdominal sopra-pubic US completed with Doppler ultrasonography, with the evidence of a pelvic mass. Two patients with acute pelvic pain underwent urgent abdomino-pelvic CT. Finally all the patients underwent MRI.

CT examinations were performed using a 64-detector-row scanner. After plain scanning, images were acquired at 30s-60s-90s after contrast medium administration.

MR exams were performed on 6 h fasting patients, pre-medicated with Hyoscine-N-butylbromide antispasmodic agent, via 1.5 superconductive magnet (Excelart Vantage, Toshiba Medical System, Japan) with phased array surface coil. The sequences included followed the ESUR recommendations for MRI of indeterminate adnexal masses [11]. The contrast-enhanced T1w Fast Spoiled 3D Fat Sat gradient-echo were dynamically acquired after iv injection of Gadobenate dimeglumine (Gd-DTPA, 0.1 mmol/Kg, injection speed 3 ml/sec) on axial, sagittal and coronal plans. Dynamic sequences were obtained at 30,60,90 and 120 s after the injection of Gd-DTPA.

MR images were retrospectively evaluated separately by two radiologists with 15 years of pertinent clinical experience in female imaging, who were blinded to clinical, histopatological, endoscopic and other imaging findings.

The following characteristics were collected: maximum diameter of the neoplasm (cm), morphology (roundish, ovoidal, lobulated) internal structure (solid, cystic, mixed cystic-solid), the presence of capsule, interlobular septa, papillary projections, parietal irregularities, calcifications (yes/no). The lesion signal intensity was compared to that of muscle tissue and judged as hypo, iso or hyper-intense and homogeneous or heterogeneous. Endolesional areas of hypo-intensity on T1-weighted images(T1w) and hyper-intensity on T2w (pure fluid signal) were classified as cystic. Areas of iso-intensity on T1w and mild hyper-intensity on T2w were classified as solid. Areas of hyper-intensity on T1w, although punctiform, with a corresponding hyper or hypointense signals on T2w, were classified as hemorrhagic.

The analysis included the evaluation of DWI signal with corresponding ADC values and the enhancement patterns of the mass and septa on T1w contrast enhanced images. Furthermore, the invasion of adjacent structures, lymphnodes number and size and the presence of endoperitoneal fluid effusions were evaluated.

Proper surgically resected specimens were submitted to an accurate hystological and cytological examination performed by a gynecologic pathologist.

## Results

All the neoplasms were unilateral. The mean diameter of the lesions was 11.9 cm (3.8-23.5 cm).

**Fig. 1:**
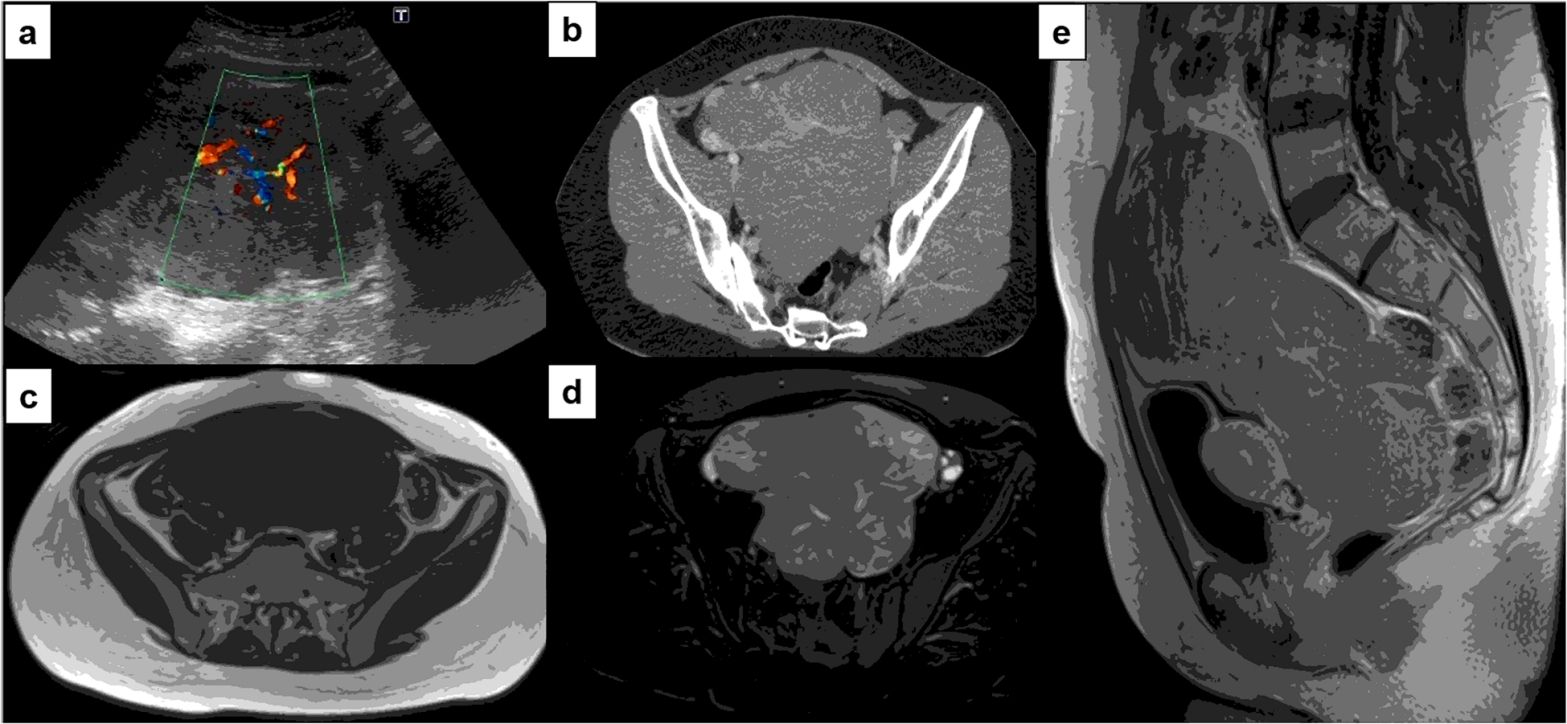
Ovarian dysgerminoma in a 19-year-old woman. Color-Doppler US images **(a)** show a hypervascular solid ovarian mass. Axial contrast-enhanced CT **(b)** shows a large-sized multilobulated right ovarian mass with enhancing septa within the lobules. Axial T1w **(c)**, axial Fat Sat T2w **(d)**, sagittal contrast-enhanced T1w **(e)** MR images show a well-marginated giant multilobulated mass in the right ovary, isointense to the muscle tissue on T1w, with hyper-intense lobules and hypo-intense septa on T2w. After contrast administration a progressive enhancement of the septa is observed

At US examinations, lesions appeared as solid masses with non-homogeneous eco-structure, with multiple endolesional hyperechoic components and vascularized septa. At CT scan the masses appeared as solid, lobulated, isodense to muscle tissues, with a moderate enhancement of wall and of endolesional fibrovascular septa (Fig. 1).

The morphology of the lesions was lobulated in 5 patients (62.5%), roundish in 2 patients (25%) and ovular in one patient.

The internal structure of the neoplastic masses was considered purely solid in 7 patients (87.5%) and mixed cystic-solid in one patient (12.5%). In 6 patients (75%), both interlobular fibrous septa and an identifiable thin and regular capsule were observed.

**Fig. 2:**
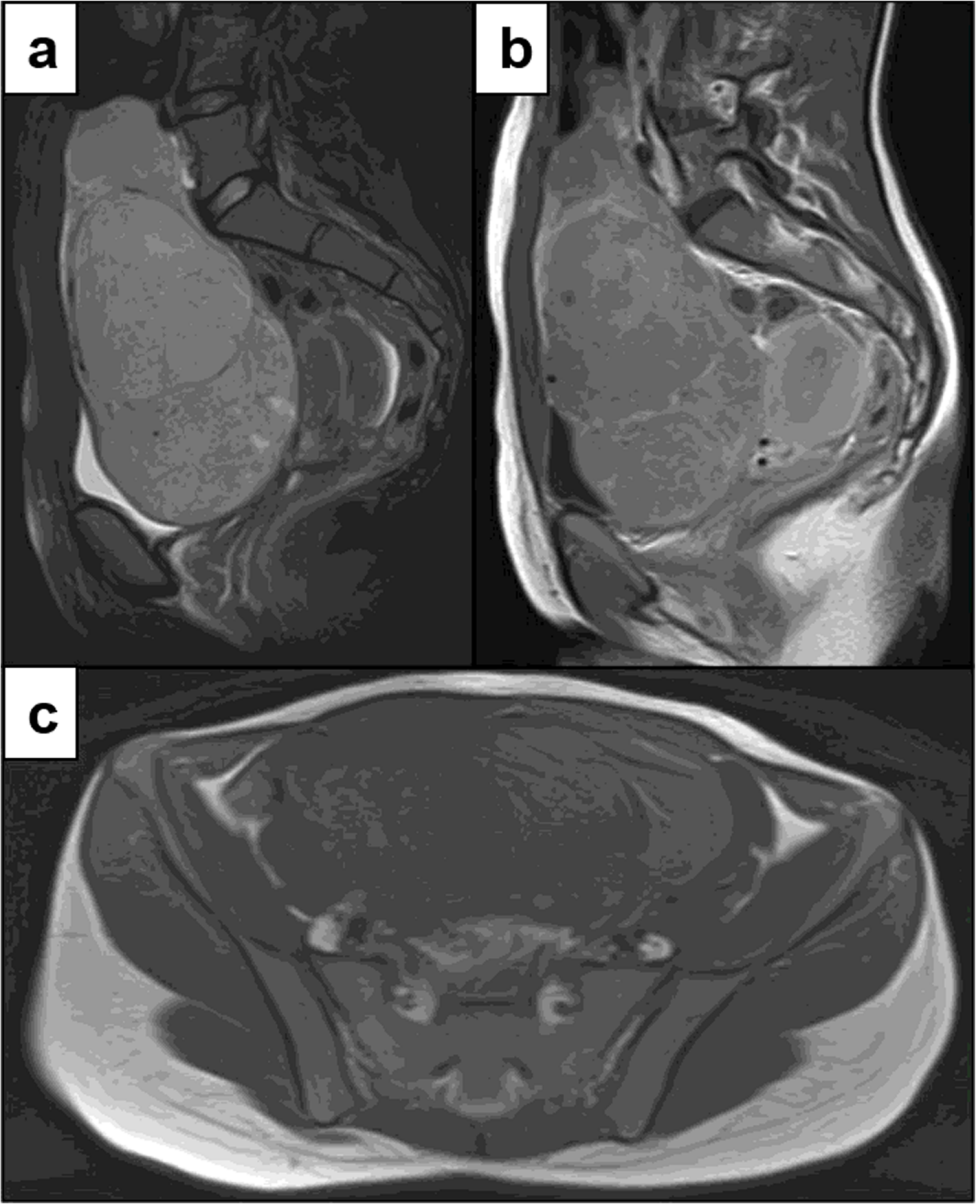
Ovarian dysgerminoma in a 20-year-old woman. Sagittal Fat Sat T2w **(a),** sagittal contrast-enhanced T1w **(b)**, axial T1w **(c)** MR images show a capsulated giant multilobulated mass displacing both bladder and uterus, isointense to the muscle tissue on T1w, with multiple lobules hyper-intense on T2w, separated by thin, regular, hypo-intense septa with avid enhancement after Gd administration. No papillary projection, parietal irregularities or calcifications are noted

No solid papillary mural projections, parietal irregularities or calcifications were detected (Fig. 2).

MR signal of the lesions was characterized by a predominant iso-intensity on T1w sequences and a moderate hyper-intensity on T2w (*n* = 8 pts.,100%), suggesting a solid appearance. On T2w sequences, septa were clearly identifiable as linear hypo-intensities in 6 (75%) cases. In 6 lobulated masses (75%) the signal was described as homogeneous; in 2 cases (25%) as heterogeneous.

**Fig. 3:**
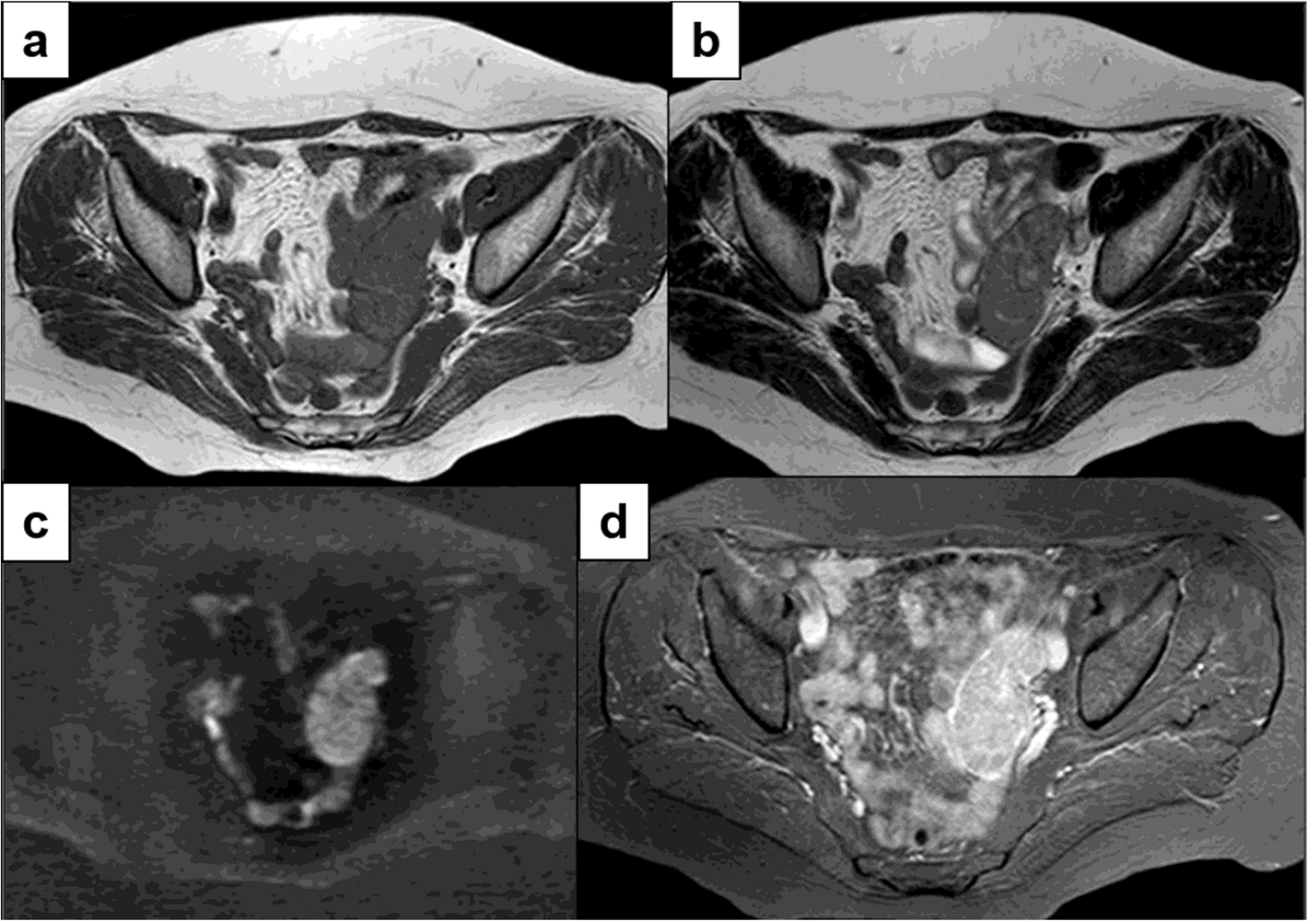
Ovarian dysgerminoma in a 34-year-old woman. Axial T1w (a), axial T2w (b), axial DWI (c), axial contrast-enhanced T1w with fat-suppression (d). MR images show an ovoidal, lobulated left ovarian mass, isointense on T1w, with heterogeneous predominantly hyper-intense signal on T2w, a restricted diffusion signal on DWI and an avid enhancement after Gd administration, with interlobular septa

Heterogeneous lesions showed hemorrhagic foci visible as focal hyper-intensity on T1w and hyper-intensity on T2w in one patient and cystic lacunae (hypo-intensity on T1 and hyper-intensity on T2w) in the other one. Mean ADC values, calculated from three b-values (0, 500,1000 s/mm^2^) on the solid areas were 0.81 × 10 - 3 mm^2^ (minimum 0.92, maximum 1.32). High signal intensity within the solid component on DWI and low signal intensity in corresponding ADC maps were observed in 8 patients (100%) (Fig. 3).

**Fig. 4:**
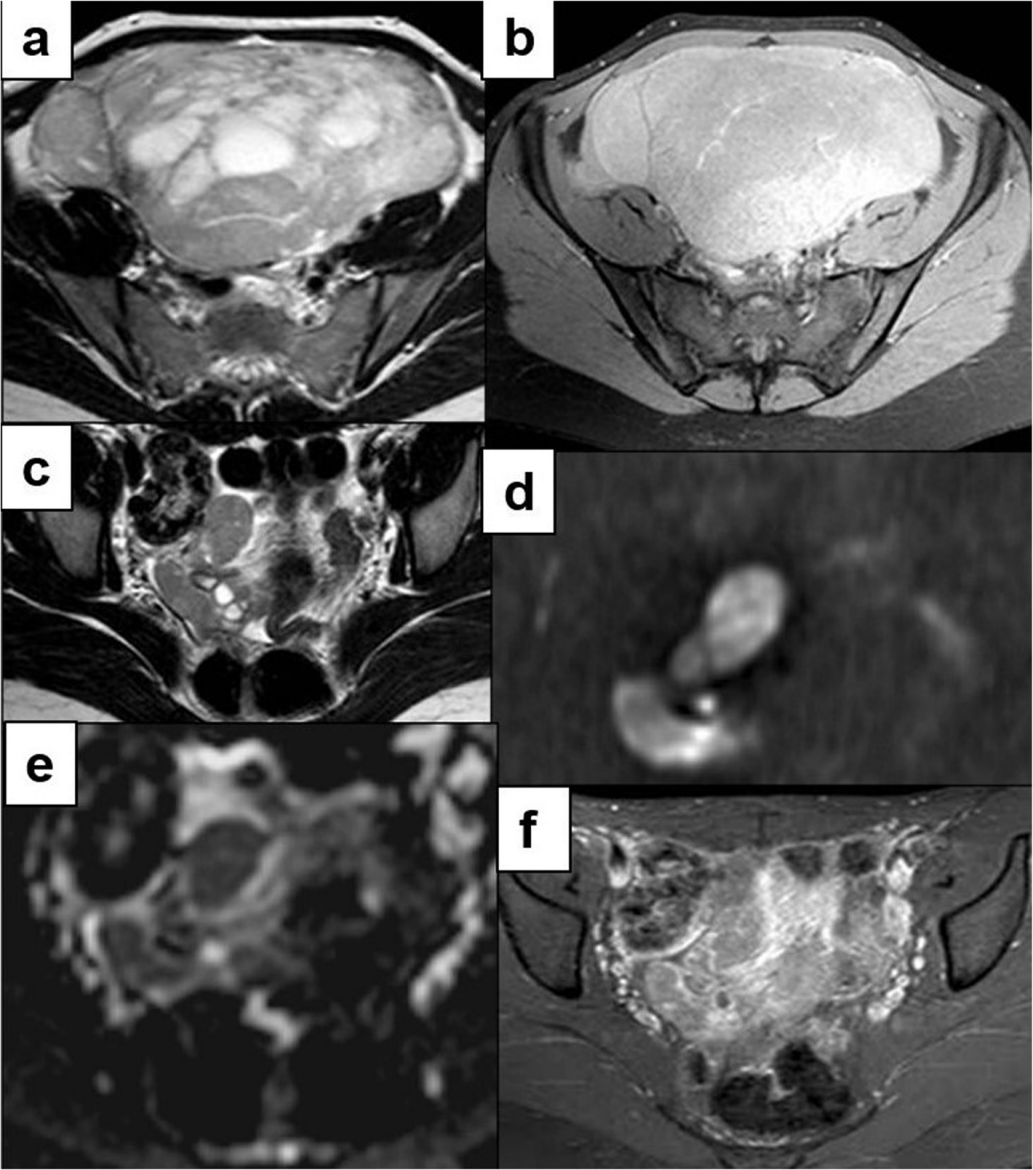
Ovarian dysgerminoma in a 23-year-old woman with recurrence after surgery. Axial T2w **(a)**, axial contrast-enhanced T1w with fat-suppression **(b)** MR images show a multilobulated heterogeneous large-sized cystic-solid pelvic mass with interspersed septa and avid enhancement after Gd administration. The mass was surgically removed and stage II dysgerminoma was diagnosed. Two years after surgical treatment, during a routine follow-up, we revealed a recurrence in the right emipelvis, characterized by heterogeneous hyperintense signal on axial T2w **(c)**, high signal and low values on DWI and ADC images **(d, e)**, heterogenous moderate enhancement after Gd administration **(f)**

After Gadolinium administration, a progressive enhancement of the fibrovascular septa and of the lobules was detected in 7 patients (87.5%) (Fig. 4). No contrast enhancement was detectable in the remaining patient.

In 2 cases (25%), a small amount of fluid effusion was observed in recto-uterine Douglas pouch.

In 2 patients (25%) lymph-nodes with short axis > 1 cm were documented in the right groin and in the left internal iliac region, respectively.

MRI findings are reported in Table 2.

**Table 2:** MRI findings

Patients (n)	1	2	3	4	5	6	7	8
**Maximum** **Ø (cm)**	5.5	3.8	23.5	14.2	10	16.8	9.7	12
**Morphology**	lobulated	roundish	lobulated	ovoid	lobulated	lobulated	roundish	lobulated
**Structure**	solid	solid	solid	solid	solid	solid	cystic-solid	solid
**Capsule**	thin	thin	no	no	thin	thin	thin	thin
**Papillary** **Projections** **Parietal** **irregularities**	no	no	no	no	no	no	no	no
**Calcifications**	no	no	no	no	no	no	no	no
**Septa**	yes	yes	yes	yes	no	yes	no	yes
**T1w signal**	iso with foci hyper	iso	iso	iso	iso	iso	iso	iso
**T2w signal**	hyper	hyper	hyper	hyper	hyper	hyper	hyper with cystic lacunae	hyper
**DWI /ADC**	high SI DWI low ADC	high SI DWI low ADC	high SI DWI low ADC	high SI DWI low ADC	high SI DWI low ADC	high SI DWI low ADC	high SI DWI low ADC	high SI DWI low ADC
**Septa/lobules enhancement**	progressive	progressive	progressive	progressive	no	progressive	progressive	progressive
**Associated findings**	inguinal lymph-nodes^a^		peritoneal effusion			internal iliac lymph-nodes^a^	peritoneal effusion	

^a^*short axis ≥ 1cm*

## Discussion and conclusions

Radiological aspects of ovarian dysgerminoma have poorly discussed in current literature. The largest multicenter radiological series have evaluated sonographic characteristics of dysgerminoma in 21 patients [7].

MR features of dysgerminoma were previously described in small series as solid masses divided into lobules by hypointense or isointense on T2w fibrovascular septa, which showed a marked enhancement after Gd-DTPA injection [8–10]. Tsuboyama et al. described three cases of non-pure dysgerminomas coexisting with other OMGCTs, being the only study reporting the presence of a high DWI signal in one case of ovarian solid mass [10]. Our series of 8 patients is one of the largest in literature including mutiparametric MRI findings and histological confirmation of pure dysgerminomas.

In the most of our cases dysgerminoma appeared as a purely solid encapsulated mass with hyper-intensity of lobules and linear hypo-intensity of septa on T2w imaging. This sign reflects the hyper-cellularity of the lobules and the fibrous nature of the septa and may help to differentiate dysgerminomas from other OMGCTs [12, 13]. The only case of mixed cystic-solid mass was noticed in an extra-ovarian (stage II) disease with a two-years cancer recurrence after radical surgery. This finding may suggest a more malignant behavior of heterogeneous mixed cystic-solid lesions compared to the purely solid ones. Contrast enhanced images may also help in differentiating dysgerminomas from other OMGCTs. In the lesions of our series, progressive enhancement of fibrovascular septa and a moderate enhancement of the lobules was observed. The other OMGCTs may also show an hypervascular behaviour although with an heterogeneous and messy structure [14, 15].

Our findings may also contribute to differentiate dysgerminomas from solid non-OMGCTs tumors including fibrotecoma, Brenner’s tumor and granular cell tumor. The first two are hypo-intense on T2w and the latter is frequently characterized by hemorrhagic endolesional areas, hyper-intense on T1w images [15]. Moreover, hemorragic foci in dysgerminoma are less common than in solid portion of other OMGCTs such as Yolk sack tumor, nongestational choriocarcinoma and embryonal carcinoma. Massive necrosis, endolesional calcifications and “ovarian vascular pedicle” sign were not detected in our cases, this latter present in up to 92% of lesions of Zhao et al. series and mostly due to thickened and tortuous ipsilateral ovarian vein [13]. The lack of endolesional calcifications and necrotic foci may also help in the differential diagnosis with epithelial carcinomas.

High signal on DWI within the solid mass with corresponding low ADC values was reported in all our cases and reflect the hypercellular environment. Nevertheless, high DWI signal and low ADC values may be observed in false-positive non-malignant lesions with high cellular density [16]. Thus, DWI should always be combined with morphological sequences.

The present study has some limitations, such as the retrospective design, that however represents a limitation typical for this very rare pathology, the small number of patients and the subjective interpretation of images.

In conclusion, our series is one of the largest available in literature including only one pure subtype of OMGCT, which is in its own very rare, analyzed by the same group of radiologists and pathologists and collected in a single center. In our series, multiparametric MR imaging is able to express its full potential in the detection and characterization of ovarian masses. Furthermore, MRI may contribute to differentiate dysgerminoma from others OMGCTs, solid non-OMGCTs and epithelial ovarian tumors. Concurrently, MRI plays an important role in local and extra-ovarian staging of dysgerminoma, in the preoperative planning and in the detection of recurrencies.

## Data Availability

The authors confirm that the data supporting the findings of this study are available within the article and its supplementary materials.

## Abbreviations

OMGCT: Ovarian malignant germ cell tumor
non-OMGCTs: Non-germ cell ovarian malignant tumor
CT: Computed Tomography
MRI: Magnetic Resonance Imaging
T1w: T1 weighted image
T1w: T2 weighted image
